# Robust estimation of group-wise cortical correspondence with an application to macaque and human neuroimaging studies

**DOI:** 10.3389/fnins.2015.00210

**Published:** 2015-06-11

**Authors:** Ilwoo Lyu, Sun H. Kim, Joon-Kyung Seong, Sang W. Yoo, Alan Evans, Yundi Shi, Mar Sanchez, Marc Niethammer, Martin A. Styner

**Affiliations:** ^1^Department of Computer Science, University of North CarolinaChapel Hill, NC, USA; ^2^Department of Psychiatry, University of North CarolinaChapel Hill, NC, USA; ^3^Department of Biomedical Engineering, Korea UniversitySeoul, South Korea; ^4^R&D Team, Health and Medical Equipment Business, Samsung ElectronicsSuwon, South Korea; ^5^Montreal Neurological Institute, McGill UniversityMontreal, QC, Canada; ^6^Department of Psychiatry and Behavioral Sciences, Emory University School of Medicine, Emory universityAtlanta, GA, USA; ^7^Biomedical Research Imaging Center, University of North CarolinaChapel Hill, NC, USA

**Keywords:** group-wise registration, cortical surface, spherical harmonics, entropy minimization, sulcal curve, surface registration

## Abstract

We present a novel group-wise registration method for cortical correspondence for local cortical thickness analysis in human and non-human primate neuroimaging studies. The proposed method is based on our earlier template based registration that estimates a continuous, smooth deformation field via sulcal curve-constrained registration employing spherical harmonic decomposition of the deformation field. This pairwise registration though results in a well-known template selection bias, which we aim to overcome here via a group-wise approach. We propose the use of an unbiased ensemble entropy minimization following the use of the pairwise registration as an initialization. An individual deformation field is then iteratively updated onto the unbiased average. For the optimization, we use metrics specific for cortical correspondence though all of these are straightforwardly extendable to the generic setting: The first focused on optimizing the correspondence of automatically extracted sulcal landmarks and the second on that of sulcal depth property maps. We further propose a robust entropy metric and a hierarchical optimization by employing spherical harmonic basis orthogonality. We also provide the detailed methodological description of both our earlier work and the proposed method with a set of experiments on a population of human and non-human primate subjects. In the experiment, we have shown that our method achieves superior results on consistency through quantitative and visual comparisons as compared to the existing methods.

## Introduction

In neuroimaging studies, group analysis of local, cortical properties is a key step to investigate disease patterns, brain growth, and group variability (Rosas et al., 2008; Chouinard-Decorte et al., 2014; Zielinski et al., 2014). A prerequisite for such an analysis is to develop consistent cortical correspondence across a population of cortices. However, the cortical folding patterns in human and non-human primates are both complex as well as highly variable across subjects. This complexity and variability yield a significant challenge to the establishment of inter-subject cortical correspondence.

Due to its high inter-subject variability, for consistent cortical correspondence it is critical to choose invariant anatomical/geometric features. With an inclusion of anatomical characteristics, anatomical landmarks often provide well established correspondence with little ambiguity, as compared to the intrinsic geometric properties such as local curvature on the cortex. In this sense, sulcal fundic regions are relatively invariant and stable across a population so have been widely used as robust features for cortical registration. Several studies also proposed sulcal fundic region recognition in recent studies (Mangin et al., 2004; Lyu et al., 2010; Seong et al., 2010), which is further employed as critical features on cortical correspondence in several articles (Thompson and Toga, 1996; Durrleman et al., 2007; Lui et al., 2010; Joshi et al., 2012; Auzias et al., 2013; Tsui et al., 2013; Lyu et al., 2013a).

Cortical correspondence methods can be categorized broadly into two main approaches: volume/voxel registration-based (Lee et al., 2013) and surface model-based registration (Oguz et al., 2009; Lombaert et al., 2013; Lyu et al., 2013a). As the volume-based registration is computed on the three-dimensional image grid, whereas the true structure of the cortical surface is a two dimensional manifold, volume/voxel based methods in general do not sufficiently incorporate the folding pattern of cortical regions for a localized analysis (Du et al., 2011). Therefore, localized cortical correspondence is significantly improved with a surface based registration on a cortical surface model. Surface model-based registrations can be further categorized as follows: (1) parametrized vs. non-parametrized surface representations and (2) pair-wise registration (individual registration to a template) vs. group-wise registration (across a population at once).

Cortical registration employing parametrized surface representations is the most prevalent in the field. It is based on the mapping of cortical surface into a specific parametrized space. Several mapping spaces have been proposed including planar (Auzias et al., 2013), hyperbolic (Tsui et al., 2013) or spherical (Tao et al., 2001; Joshi et al., 2007) parametrizations. Spherical parametrizations are most popularly used due to their convenience, reduced distortions and computational efficiency (Thompson and Toga, 1996; Fischl et al., 1999; Yeo et al., 2010; Park et al., 2012; Robinson et al., 2013; Lyu et al., 2013b). To reduce mapping distortions in parametrization, geometric features (e.g., local curvatures, curveness, shape index, etc.) have been widely employed in popular pipelines such as Freesurfer. In recent work (Lui et al., 2010; Auzias et al., 2013; Tsui et al., 2013), sulcal landmark features were employed to reduce such mapping distortion. Alternatively, Shi et al. (2013) proposed an embedding in the Laplace-Beltrami (LB) space that incorporates spectral representation to reduce distortions. While these parametrization-based methods are able to provide appropriate parametrized representations, it is noteworthy that all such parametrizations possess significant residual mapping distortions.

Several researchers proposed cortical surface registration in a non-parametrized space. Non-parametric cortical representation is advantageous in that no cortical mapping is required to avoid distortion of the original surface representation. A spectral-based approach is applied to represent shape descriptors of a cortex by solving the Eigenfunctions of the LB operator, which provides an intrinsic features for cortical surface matching in the spectral domain (Niethammer et al., 2007; Lombaert et al., 2013). In Cates et al. (2007); Oguz et al. (2009), particle-based registration is applied to cortical surface models on which particles are spread and located to establish a correspondence across subjects. Though these non-parametric methods are free from mapping distortion as surface model representation, it is difficult to establish continuous correspondence without interpolation of the discrete deformation field.

Template-based methods have been well studied that establishes a correspondence with a template model in a pair-wise manner. A spherical mapping to the template space is a well known and widely used approach of cortical registration. Several studies (Thompson and Toga, 1996; Fischl et al., 1999; Yeo et al., 2010; Park et al., 2012) have shown pair-wise registration in the spherical space that allows every subject to be aligned to a single template model. Van Essen (2004) applied a surface registration method to human and even non-human primate subjects via spherical mapping. Lyttelton et al. (2007) further proposed an iterative registration scheme that updates the initial template model for better correspondence establishment. We also proposed cortical registration via spherical harmonic decomposition of the deformation field (Lyu et al., 2013a). Unfortunately, even if individual correspondence to the template is well developed, these pair-wise methods are difficult to reduce an inherent bias to the initial template model, which is less preferable for our goal that yields better sensitivity and specificity in statistical analysis on a population.

As reported in Styner et al. (2007); Oguz et al. (2008), group-wise correspondence methods generally yield better statistical shape models in that cortical correspondence is established at once during registration. In earlier work (Davies et al., 2003; Twining et al., 2005; Styner et al., 2008; Davies et al., 2010), a minimum description length (MDL) scheme is applied to describe shape models across a population in a group-wise manner. Also, Cates et al. (2007) adapted entropy minimization akin to MDL to formulate particle-based registration on cortical surface models without using a template model or prior information. Indeed, MDL is equivalent to entropy minimization as revealed in Kotcheff and Taylor (1998); Cover and Thomas (2012). Oguz et al. (2009) further refined the particle-based registration with incorporation of curvature features, showing the improved correspondence via the analyses of cortical thickness over the entire cortical surface. However, a particle-based correspondence implicitly defines a deformation model without guarantee of topology preservation. In other words, their method is likely to yield folding on the cortical surface after registration. These methods were difficult to provide an explicit estimation of a deformation field between subjects.

Our work presented here is based on our previous pair-wise cortical correspondence method using spherical harmonic decomposition (Lyu et al., 2013a), where we use spherical harmonic decomposition to continuously represent a smooth deformation field over the sulcal landmarks and sulcal depth maps. Since pair-wise registration is potentially of template bias, group-wise registration is expected to have better correspondence in group-wise analysis purpose such as cortical thickness.

This proposed group-wise approach has been initially presented at a conference (Lyu et al., 2013b) and is extended here in the following ways: (a) we propose robust entropy estimation to reduce the influence of landmark extraction and labeling errors, (b) we accelerated the computation by employing orthonormality of the harmonic basis functions, (c) a novel surface coloring for visual comparison, (d) provide additional experimental results for validation, and (4) more experiments with respect to robustness and comparisons with the existing methods.

## Methods

**Figure 1:**
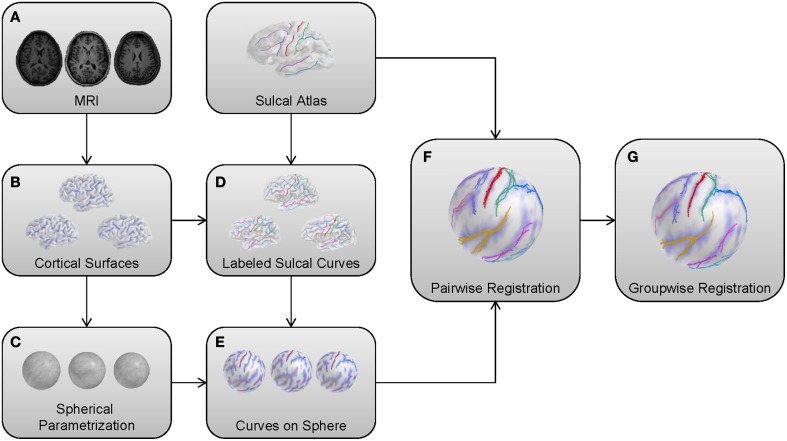
**Schematic overview of the proposed method. (A)** MRIs as input models. **(B)** Cortical surfaces reconstructed from a surface reconstruction pipeline. **(C)** Reconstructed surfaces with a spherical parametrization. **(D)** Automatic sulcal curve extraction and labeling. **(E)** A spherical mapping of labeled sulcal curves. **(F)** Pair-wise registration to the template for individual correspondence. **(G)** Group-wise registration for unbiased estimation.

In this section, we adapt our earlier method as described in Lyu et al. (2013a). We apply the pairwise registration without modification, but extend the group-wise registration for robust cortical correspondence estimation by introducing modified terms employed in entropy computation. Briefly, the overall framework of the proposed method is summarized as follows: (1) Cortical surfaces are reconstructed from MRIs with spherical parametrization. (2) Sulcal curves are automatically extracted with their labels and mapped onto the sphere. (3) Pair-wise registration is performed on the mapped sphere (common space) to establish an individual correspondence to the template. (4) Group-wise registration is applied to the pair-wise correspondence for unbiased estimation regardless of the template choice. Figure 1 illustrates a schematic overview of our pipeline.

### Landmark correspondence

We use automatic sulcal curve extraction (Seong et al., 2010) and automatic sulcal curve labeling (Lyu et al., 2010) to extract/label a separate set of labeled sulcal curves. In particular, the unlabeled sulcal curves consisting of ordered sets of points without branching are extracted from the triangulated surface by the sulcal curve extraction method. Thus, no branching is taken into account, though each sulcal curve can be (and is often) composed of multiple separated curve segments. Then pre-labeled sulcal curves (according to the protocol in Lyu et al., 2013a for the macaque subjects and Lyu et al., 2010 for the human subjects) are employed to label matched (corresponding) unlabeled sulcal curves, while discarding minor and extraneous curves. This labeling method further establishes a point-by-point correspondence on these sulcal curves called sulcal landmarks in the remainder of this paper.

### Sulcal curve-constrained pair-wise correspondence

#### Problem definition

For two given triangulated cortical surfaces (template and subject), we denote *V*_temp_ and *V*_subj_ as the template and subject surfaces. Our goal is to estimate a continuous cortical correspondence *M*: ℝ^3^ → ℝ^3^ such that

(1) u=M(v) ,

where locations *v* on the subject surface *V*_subj_ are mapped to the corresponding locations *u* on the template surface *V*_temp_.

#### Consistent displacement encoding scheme

To take advantage of the well known spherical parametrization, we use an invertible spherical mapping ψ(·): ℝ^3^ → ℝ^2^ established in the preprocessing stage. We thus map all vertices of the cortical surfaces onto the common unit sphere, which reduces our correspondence estimation to find *M*: ℝ^2^ → ℝ^2^. This spherical mapping establishes an initial cortical correspondence, which is further improved in the proposed method.

**Figure 2:**
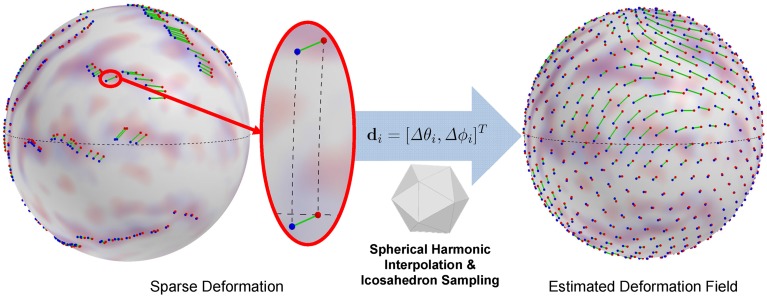
**Displacement encoding and estimated deformation field (an icosahedron sampling of continuous representation)**. A spherical displacement is encoded as change in spherical angles after rotation onto the equator for arclength preservation, which avoids distorted displacement representation. The interpolation of the deformation field is obtained by spherical harmonic decomposition.

We then locally encode the deformation as a displacement in local spherical polar angles of elevation Δθ and azimuth Δϕ. It is well-known that the local spherical angles at different locations on the sphere based on a single frame of reference (spherical coordinate system) yields inconsistent representations of the same geodesic length. For example, a given displacement (Δθ, Δϕ) at the equator yields a longer geodesic arc-length than the same displacement closer to the pole. To avoid such an inconsistency and thus to provide an arclength consistent encoding, we adapt a locally normalized polar system. Let *p* and *q* be corresponding landmarks from a subject and the template, respectively. First, we find a rotation matrix **R**_*p*_ with an angle (≤ 90°) along the longitude circle passing through *p* and the two poles, such that *p* is exactly located on the equator. By applying **R***_p_* to *p* and *q*, we then compute the normalized polar displacement vector Δθ and Δϕ. Thus, the local landmark displacement at spherical vertex *i* (θ_*i*_, ϕ_*i*_) on the unit sphere is represented as a vector **d***_i_* = ψ(**R**_*p_i_*_ · *p_i_*) − ψ(**R**_*p_i_*_ · *q_i_*) = [Δθ*_i_*, Δϕ_*i*_]^*T*^ (see Figure 2).

#### Initial deformation field

To find an initial deformation field of the entire surface, we compute least squares fitting of spherical harmonic basis functions to displacements of sulcal landmarks established in the sulcal labeling step. This fitting is standard spherical harmonic decomposition of the [Δθ_*i*_, Δϕ_*i*_] spherical signal. At a point (θ, ϕ) on the sphere, the spherical harmonic basis functions with degree *l* and order *m* (−*l* ≤ *m* ≤ *l*) are given by

(2) $$Y_{l}^{m}(\theta ,\phi )=\sqrt{\frac{2l+1}{4\pi }\frac{(l-m)!}{(l+m)!}}P_{l}^{m}(\cos\theta )e^{im\phi }\;,$$

(3) $$Y_{l}^{-m}(\theta ,\phi )={\left(-1\right)}^{m}Y_{l}^{m*}(\theta ,\phi )\;,$$

where *Y*^m*^_l_ denotes the complex conjugate of *Y^m^_l_* and *P^m^_l_* is the associated Legendre polynomial

(4) $$P_{l}^{m}(x)=\frac{{\left(-1\right)}^{m}}{2^{l}l!}{\left(1-x^{2}\right)}^{\frac{m}{2}}\frac{d^{\left(l+m\right)}}{dx^{\left(l+m\right)}}{\left(x^{2}-1\right)}^{l}\;.$$

Since the basis functions are defined in the complex domain, we use a real form of the functions defined by

(5) $$Y_{l,m}=\{\begin{array}{ll} \frac{1}{\sqrt{2}}(Y_{l}^{m}+{\left(-1\right)}^{m}Y_{l}^{-m}) & m>0\;,\\ Y_{l}^{0} & m=0\;,\\ \frac{1}{\sqrt{2}i}(Y_{l}^{-m}-{\left(-1\right)}^{m}Y_{l}^{m}) & m<0\;. \end{array}$$

Given degree *l* of spherical harmonic decomposition, we assume that the number *n* of the landmarks is larger than the dimension of (*l* + 1)^2^ spherical harmonic basis functions to prevent a rank deficient problem. The coefficients can then be estimated by standard least squares fitting.

(6) $$C={\left(YY^{T}\right)}^{-1}YD^{T}\;,$$

where **D** = [**d**_1_, **d**_2_, …, **d***_n_*] and **Y** is a (*l* + 1)^2^ by *n* matrix that incorporates the spherical harmonic bases. Once the coefficients of the spherical harmonic decomposition are computed, for a point *v* ∈ *V*_subj_ subject space, its deformed position in the template space is easily reconstructed by the spherical mapping function $\hat{M}$.

(7) $$\hat{u}=\hat{M}(v)=R_{v}^{T}\cdot \psi^{-1}(\psi (R_{v}\cdot v)+C^{T}\cdot Y_{v})\;,$$

where **Y**_*v*_ is a column vector of the spherical harmonic basis at ψ(v) and **R***_v_* is a rotation matrix that puts *v* on the equator. Figure 2 shows an example of the estimated deformation field.

The basis functions are linearly independent due to their orthogonal property. We employ the hierarchy in that the initial deformation field is computed via low degree (*l* = 5) fitting of the sulcal landmarks and higher degree representations are used in the optimization stage.

#### Optimization

As discussed in the earlier section, the initial coefficients are determined only by sulcal landmarks, which biases the cortical correspondence to the specific sulcal fundic regions selected in the sulcal labeling step and affected by minor mislabeling errors. For improved correspondence establishment, we further formulate a metric that incorporates sulcal landmark errors and agreements between sulcal depth maps via normalized cross correlation (NCC) over the entire cortical surface. To regularize the impact of landmark errors, we define an M-estimator based weighting function *f* under Gaussian assumption in Equation (8). By incorporating *d*_min_ as voxel size, landmark errors are ignored if below *d*_min_ distance and reduced to a maximal contribution if over a maximum distance *d*_max_. We chose *d*_max_ about 10–20 times larger than *d*_min_ based on experimental observations.

(8) $$f(d)=2\int_{d_{\text{min}}}^{d}\frac{I(d)}{\sigma \sqrt{2\pi }}\exp\left\{-\frac{1}{2}{\left(\frac{x-d_{\text{min}}}{\sigma }\right)}^{2}\right\}dx\;,$$

(9) $$I(d)=\{\begin{array}{ll} 1 & \;d\geq d_{\text{min}}\;,\\ 0 & \text{ otherwise }, \end{array}$$

where 6 · σ = *d*_max_ − *d*_min_. Now, we define *L*(·, ·) = *f*(η·arclen(·, ·)) as a regularized arclength, where η is a ratio of the geodesic distance between two points mapped on the unit sphere and on the template surface. Practically, it can be approximated as a ratio of the triangle size under the assumption that the template surface consists of uniform triangles. The resulting overall cost function is thus formulated with a regularization factor *w* by letting an operator ⊗ denote normalized cross correlation between two sulcal depth maps.

(10) $$\begin{array}{l} \hat{C}=\arg\underset{\text{C}}{\min}[w\left\{\frac{1}{n}\sum_{i=1}^{n}L(p_{i},\hat{p_{i}})\right\}\\ \;+(1-w)\left\{\frac{1}{2}\left(1-S(\{u\})⊗S(\{\hat{u}\})\right)\right\}], \end{array}$$

where *p*_*i*_ and $\hat{p_{i}}$ are two corresponding sulcal points and *S*(·) is a sulcal depth map reconstructed from a set of vertices. The optimization procedure employs the NEWUOA optimizer (Powell, 2006) for minimizing $\hat{C}$, which finds an optimal solution without derivatives. In our experiments, we empirically set *w* = 0.5 based on the experiments in Lyu et al. (2013a).

#### Optimal pole selection

**Figure 3:**
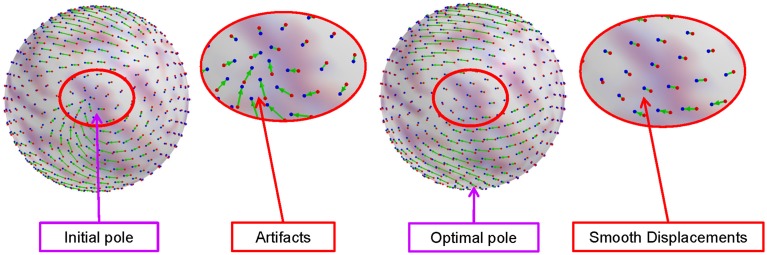
**Artifacts in the standard polar coordinate system and reduced artifacts (smooth deformation field) by the optimal pole selection**. The artifacts introduced by spherical harmonic interpolation are significantly reduced by choosing a proper pole.

The direction of displacements are variant to location in the proposed spherical polar coordinate system. Depending on rotation to the equator, two identical displacements have a different sign in polar angles if they are computed on opposite sites with respect to the poles. This can yield a deformation field with significant distortions leading to even sign changes close to the poles. Therefore, a proper choice of the pole **ê** can significantly minimize this influence and yield most smooth deformation fields. In our observation, the presence of non-smooth deformations generally leads to high magnitude coefficients in high frequency bases. Thus, we define a coefficient sum-based metric that weighs higher frequency coefficients stronger.

(11) $$\hat{e}=\arg\underset{{}_{e}}{\min}\sum_{l=0}^{k}\sum_{m=-l}^{l}\left(l+1\right)\cdot \{|c_{\theta_{l,m}}|+|c_{\phi_{l,m}}|\}\;,$$

where *c*_θ_ and *c*_ϕ_ are coefficients for elevation and azimuth displacements, respectively. As this metric possibly has local minima, we initialize the optimization with multiple initial guesses spread across the sphere and select the overall minimum as the final pole selection. Figure 3 shows an example of artifacts in the standard polar coordinate system, while significantly reduced artifacts by optimal pole selection.

### Extension to group-wise correspondence

**Figure 4:**
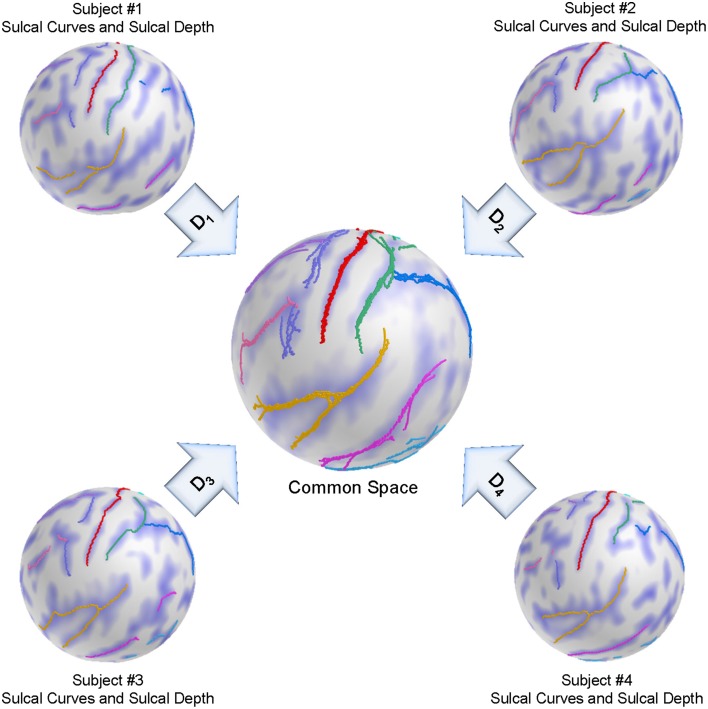
**Schematic overview of the group-wise registration**. An initial correspondence for each subject is established by our pair-wise registration. The deformed sulcal curves and depth maps are mapped onto the sphere based on the pair-wise correspondence. The group-wise correspondence is then iteratively updated across a population via entropy minimization.

We adapt here a group-wise registration to further improve pairwise registration results as well as to remove the template selection bias inherent to pairwise registration as described in Lyu et al. (2013a). A group-wise correspondence is computed independently from the template and thus is expected to perform more stably across a population of surfaces. The group-wise correspondence method incorporates modified entropy terms computed over the landmark distributions and sulcal depth maps. Figure 4 shows a schematic overview of the group-wise registration.

#### Problem definition

For *N* given triangulated cortical surfaces mapped onto the unit sphere, each of which has the same number *n* of the common corresponding vertices, we let *V^i^* be the *i*th surface, *i* = 1, …, *N*. The goal is to estimate continuous mapping functions of cortical correspondence *M*_*i*_: ℝ^2^ → ℝ^2^ that map surfaces into a common reference space such that

(12) $$M_{1}(v^{1})=M_{2}(v^{2})=\cdots =M_{N}(v^{N})\;,$$

where *v^i^* are the corresponding locations on the subject surface. Let **x**(*M_j_*) be a column vector of the corresponding points of subject *j* deformed by *M_j_*, i.e., **x**(*M_j_*) = [*M_j_*(*v*^*j*^_1_), …, *M_j_*(*v^j^_n_*)]^*T*^. As described in Oguz et al. (2009), we assume that **x**(*M_j_*) are instances of **X** drawn from a probability density function *p*(**X**). The amount of the information in the random sampling is given by the entropy *H*[**X**], and the minimization problem is then formulated as follows.

(13) $$\{{\hat{M}}_{1},\cdots ,{\hat{M}}_{N}\}={\text{arg min}}_{\left\{M_{1},\cdots ,M_{N}\right\}}H[x]\;,$$

which drives mapped/deformed corresponding points closer to each other.

#### Entropy of landmark errors

In the previous section, we describe the pair-wise correspondence as initialization for the proposed group-wise method. As the sulcal labeling procedure yields varying parts of sulcal curves being labeled, we constrain the set of sulcal landmarks only those that have a full correspondence across all cortical surfaces. A key step for entropy computation is the density estimation of corresponding landmarks. However, appropriate density estimation on the sphere can be computationally demanding, as it involves geodesic distance computation. Similar to Oguz et al. (2009) for efficiency, we assume that the initial mapping well centralizes corresponding landmarks, which allows a mapping from the spherical space to the Euclidean space ℝ^2^ → ℝ^3^ under the assumption of the proximity of corresponding landmarks. We first compute the average over corresponding landmarks, which is rescaled to the sphere, and the landmarks are projected onto the tangential plane at that approximated mean to enable Euclidean statistics.

#### Entropy of sulcal depth

Since landmarks are sparsely distributed over the sphere and also likely possess minor mislabeling, we employ additional entropy computation over sulcal depth maps densely sampled across the surface. Let *s*(·) denote the sulcal depth at a given point and *v^j^* be the point in the *j*th subject such that *u* = *M_j_*(*v^j^*), where *u* is a given point on the sphere. Given *M_j_*, *v^j^* has a correspondence with its corresponding vertices. Ideally, there will be little difference in sulcal depth across the corresponding points if the mapping is well established, i.e., *s*(*M*_1_(*v*^1^)) ≅ … ≅ *s*(*M_N_*(*v^N^*)). By uniform icosahedron subdivision-based spherical sampling of *u*, sulcal depth agreement is straightforwardly plugged into the entropy minimization problem.

#### Entropy minimization

We model **x**(*M_j_*) as an instance of **X** such that

(14) $$x(M_{j})={\left[proj_{{\bar{v}}_{1}}(M_{j}(v_{1}^{j})),\cdots ,proj_{{\bar{v}}_{n}}(M_{j}(v_{n}^{j})),S(\{M_{j}(v^{j})\})\right]}^{T}\;,$$

where *proj*(·) denotes the projection of a vertex onto the tangential plane at the approximated mean over the corresponding landmarks. For the density estimation, we assume a multivariate Gaussian distribution with covariance Σ and therefore, the entropy is obtained by

(15) $$H[X]\approx \frac{1}{2}\ln|\Sigma |=\frac{1}{2}\sum \ln\lambda \;,$$

where λ are the eigenvalues of Σ. By letting **x** be the sample mean and **z** = [**x**(*M*_1_) − **x**, …, **x**(*M_N_*) − **x**], the sample covariance is given by $\frac{1}{N-1}zz^{T}$. In general, the dimension of **X** is much larger than *N*, the sample covariance is not fully ranked (*N* − 1). As stated in Oguz et al. (2009), we instead compute eigenvalues of $\frac{1}{N-1}z^{T}z$ in the dual space, which is fully ranked. The optimization uses the same NEWUOA optimizer (Powell, 2006) for solving the entropy cost function as in the pair-wise correspondence.

#### Robust estimation to group-wise entropy

As stated in earlier studies (Lyu et al., 2010, 2012), the extracted sulcal curves can be incorrectly identified during sulcal labeling, which can yield significantly large errors on computation of group-wise entropy over the sphere. Unfortunately, it is difficult to handle such mislabeled curves without manual modification. We instead compute the median of the corresponding points rather than the Euclidean mean generally used in the conventional entropy computation. Thus, we estimated the “corrected” mean as the median of the projected landmarks. For the *k*th landmark, the estimate is given by *v*_*k*_ = Median{*M*_1_(*v*^1^_*k*_), *M*_2_(*v*^2^_*k*_), …, *M_N_*(*v^N^_k_*)}. Note that the tangential plane is also defined by a point *v*_*k*_ that at the median of the corresponding landmarks *k*.

It is likely in the sulcal labeling method to mislabel curves as stated in Lyu et al. (2010). The mislabeled sulcal data on the plane generally leads to high entropy since they are located quite away from the median. We thus prefer to minimize influences from the mislabeled data. To achieve minimization of such influences, we define an M-estimator like a weighting function to weigh the Euclidean distances of the tangential plane similarly as in pairwise registration. We employ the same weighting function (Equations 8 and 9) for this purpose, where the large distance is less considerable during entropy computation as the landmark is highly likely to be a mislabeled one.

#### Hierarchical optimization

It can be easily observed that the computational time depends mainly on the number of landmarks and a sampling of the sulcal depth over the sphere, which is directly associated with the eigenvalue computation with respect to the size of the covariance matrix. A nice property of the spherical harmonic decomposition is orthonormality of basis functions, which makes it possible to compute coefficients in a semi-independent way for sets of basis functions. To reduce computational time, we first compute coefficients in blocks of *l* size, i.e., start with the first *l* low degrees, then the next lowest *l* degrees while fixing the already computed coefficients. Once all coefficients have been obtained this way, we finally optimize all coefficients together to further tune the coefficients to reach optimal values. We set *l* = 3 in the experiment.

### Evaluation

#### Correspondence evaluation via surface coloring

**Figure 5:**
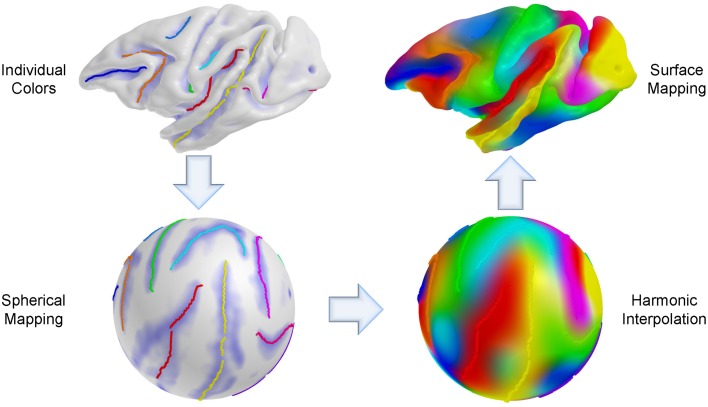
**Cortical surface coloring using spherical harmonic interpolation. An individual curve has its own color for propagation and the sulcal curves are then mapped onto the sphere**. For each RGB channel, a color intensity is interpolated with a combination of harmonic basis functions, and the color maps obtained via interpolation are remapped onto the original space (cortical surface).

**Figure 6:**
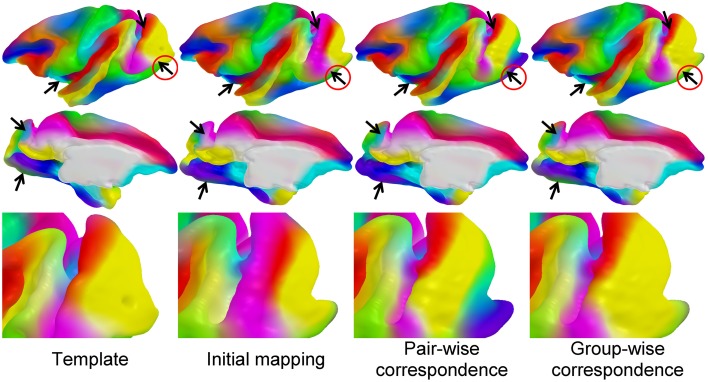
**Visual comparison of correspondence results: the colored template surface (first column) is propagated to a selected, representative example surface via initial spherical mapping (second), pair-wise correspondence (third), and group-wise correspondence (fourth)**. The arrows indicate areas of visual differences across the correspondence methods. Visually well-distinguishable regions are circled in red.

We established a sulcal curve-based color mapping in the template, which we propagate across all cortical surfaces using the established correspondence in order to provide a visual quality assessment of that correspondence. Since only few curves with distinct colors are available, we aim at propagating those colors over the entire surface. To generate the reference colorized template surface, each RGB channel was independently interpolated to the full surface via spherical harmonic decomposition on the spherical parametrization (see Figure 5). Due to no ground truth available in our experiments, this visualization allows to visually evaluate correspondence between multiple surfaces effectively, as corresponding locations are visualized with the same color. In Figure 6, the proposed group-wise method shows qualitative improvement over the pair-wise correspondence.

#### Entropy-based sulcal variability

We measured how well sulcal curves were aligned after registration through quantitative comparisons and visualization. One can use a mean curve to measure sulcal variability. However, as stated in several studies (Lyu et al., 2010; Joshi et al., 2012), such a mean curve computation is non-trivial. We instead propose a sulcal curve propagation scheme based on entropy of the sulcal points across a population. Specifically, for each major sulcus, we first put sulcal points onto the image space where all sulcal points are fully spanned, which is chosen as the same dimension of MRI resolution (e.g., 256 × 256 × 256). Then in this image space, we apply propagation from the sulcal points as seed points by adapting an isotropic level set-like scheme. This gives an Euclidean distance for each voxel that describes a distance to the closet seed point. Once the propagation is completed, we compute the average distance maps for each curve set. We compute a probability map from the average map by assuming that the distances follow a Gaussian distribution. Thus, the probability at voxel having a distance *d* is given by $\frac{1}{\sqrt{2}\pi \sigma }\exp-\frac{{\left(d-\mu \right)}^{2}}{2\sigma^{2}}$, where μ = 0 mm, and σ = 25 mm. Finally, we employ this probability map to compute the entropy over all voxels. This entropy is expected to be minimal if the curves are well aligned.

## Results

We applied the proposed method on both non-human primate and human subjects to evaluate the established correspondence quality. Since there exists no ground-truth for a cortical correspondence, we made comparisons with the initial spherical mapping and the pair-wise method via analyses on cortical thickness as well as the agreement with manually extracted sulcal curves. We also provide experiments on consistency and reliability through quantitative and visual comparisons with the existing methods.

### Dataset

For both human and non-human primate data, we randomly selected one subject as template and manually labeled its sulcal curves. Sulcal curves were labeled according to the protocol used in Lyu et al. (2013a) for the human subjects and Lyu et al. (2013a) for the macaque subjects, respectively. For visual assessment, all curves were also manually labeled on the 24 remaining subjects. In the experiment, we used the same manually labeled major curves as our previous work (Lyu et al., 2013b).

**Figure 7:**
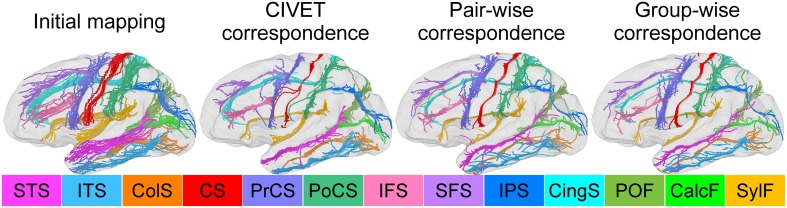
**Sulcal curve alignment by initial spherical mapping (1st column), CIVET correspondence (2nd), pair-wise correspondence (3rd), and group-wise correspondence (4th)**. The arrows indicate improved agreement in the group-wise as compared to the pair-wise correspondence.

For the human data, we delineated the 13 major sulcal curves on each left hemisphere: superior temporal (STS), inferior temporal (ITS), temporo-occipital (TOS), central (CS), precentral (PreCS), postcentral (PostCS), inferior frontal (IFS), and superior frontal (SFS), intraparietal (IPS), cingulate (CingS), calcarine (CalcS), occipito-parietal (OPS), and sylvian (SylS) sulcus (see Figure 7). We used *l* = 15 degrees of spherical harmonic basis functions. For the macaque data, we had the 9 major curves on the left hemispheres: central, arcuate, principal, superior temporal, lunate, cingulate, intraparietal, occipito-parietal, and sylvian sulcus.

#### Macaque cortical surfaces

We used 18-months-old macaques imaged under anesthesia at the Yerkes Imaging Center (Emory University, GA) on a 3T Siemens Trio scanner with an 8-channel phase array trans-receiving volume coil using MPRAGE with GRAPPA optimized to a high resolution at 0.6 × 0.6 × 0.6 mm^3^ (TR = 3,000 ms, TE=3.33 ms, flip angle = 8°, matrix = 192 × 192). The cortical surfaces were reconstructed by the CIVET pipeline (Kim et al., 2005). All experiments described in this study were performed in accordance with the NIH Guide for the Care and Use of Laboratory Animals and approved by the Emory University Institutional Animal Care and Use Committee (IACUC).

#### IBIS dataset

Pediatric 2-year-old subjects were acquired on 3T Siemens Tim Trio scanners at 1 × 1 × 1 mm^3^ resolution with T1-(160 slices with TR = 2400 ms, TE = 3.16 ms, flip angle = 8°, matrix = 256 × 256) and T2-weighted (160 slices with TR = 3200 ms, TE = 499 ms, flip angle=120°, matrix = 256 × 256) scans. 25 subjects were randomly selected from scans acquired as part of the IBIS (Infant Brain Imaging Study) network (http://www.ibis-network.org, IRB approval, Informed consent was obtained from all parents of participants for screening and evaluations) at four different sites (University of North Carolina at Chapel Hill, University of Washington at Seattle, Washington University at Saint Louis, and the Children's Hospital of Philadelphia). The cortical surfaces were reconstructed by the CIVET pipeline (Kim et al., 2005).

### Visual validation on macaque dataset

We established the cortical correspondence on the macaque dataset. In Figure 6, the proposed group-wise method shows qualitative improvement over the pair-wise correspondence.

### Sulcal curve variability

We also investigated how well/tightly sulcal curves were aligned in the group-wise common space. Note that we used only automatically labeled curves for the cortical correspondence establishment and that the manually delineated curves were employed for validation purpose only, and thus are independent of the optimization step in the proposed method. Visually, the mapped major sulcal curves showed improved agreement in several regions as compared to the pair-wise correspondence as shown in Figure 7. Our method achieved lower entropy except for ITS and PoCS as compared to that computed via CIVET as shown in Table 1. This is because ITS and PoCS labelings are more difficult to establish during automatic sulcal labeling.

**Table 1:** **Sulcal curve entropy measured based on the proposed metric**.

	**STS**	**ITS**	**ColS**	**CS**	**PrCS**	**PoCS**	**IFS**
CIVET	12.0908	12.2759	12.6610	12.2135	13.0999	11.7675	9.5944
Ours	11.9334	13.0723	11.1198	12.0140	12.6433	12.1376	7.9299
	**SFS**	**IPS**	**CingS**	**POF**	**CalcF**	**SylF**	
CIVET	12.2897	12.2183	15.9611	9.9457	14.3972	12.0675	
Ours	11.7407	11.8827	15.8695	9.6593	11.6527	11.3509	

### Variance over sulcal depth maps and cortical thickness

For quantitative evaluation on the correspondence quality, we first measured cross-subject variance estimates of sulcal depth over all vertices on the entire surface across subjects. However, such an evaluation is biased, as sulcal depth is employed in the cost function. We further used variance estimate of cortical thickness as well as visual assessment of manually labeled sulcal curves for unbiased evaluation. In Table 2, the variance analysis indicates superior performance of our method for sulcal depth and cortical thickness measures, with significant differences to both the initial mapping and the pair-wise method, revealed by Student's *t*-test (*p* < 0.0001).

**Table 2:** **Variances of cortical properties for different correspondence methods (unit: *mm*)**.

	**Depth**		**Thickness**	
	**Mean**	**Std**	**Mean**	**Std**
CIVET	1.1996	0.5103	0.4942	0.3314
Initial	2.1313	0.9938	0.5317	0.3547
Pair-wise	1.5815	0.8358	0.4996	0.3539
Group-wise	1.5568	0.8180	**0.4712**	**0.3375**

*For both sulcal depth and thickness variances, the proposed method shows significant differences against both the initial mapping and the pair-wise method (p < 0.0001). The bold indicates that our group-wise registration achieves the lowest variance of the cortical thickness*.

## Conclusion

We presented an automatic group-wise cortical correspondence method that estimates a smooth continuous deformation field using entropy minimization incorporating two terms: sulcal landmarks for local alignment and sulcal depth map for the cortical regions that are not covered by sulcal curves. To overcome potential mislabeling in the sulcal curve labeling procedure, we proposed both median estimation of the sulcal points on the tangential plane and weighted distances for reduction of mislabeling influences. To measure sulcal curve alignments, we introduced an entropy-based metric that quantifies variability in the sulcal alignment. We finally provided a detailed description of cortical surface coloring for visual comparisons.

In our experiments, the proposed method outperformed the pair-wise method in human subjects via quantitative analysis and visual comparisons as well as in non-human subjects via visual assessment. Statistical analysis also provided evidence that our method has better consistency and reliability on different dataset as compared to the existing CIVET method (*p* ≤ 0.001 in both cases). Specifically, for consistency, the proposed method achieved tighter sulcal curve alignment and sharper sulcal depth map average.

The proposed method allows an inclusion of additional clinical information such as DTI-based connectivity (Oguz et al., 2009) or myelin map alignment (Robinson et al., 2013). Furthermore, any known prior information can be straightforwardly propagated from the template to assist better cortical correspondence. Our method could further be improved by incorporating the proposed sulcal variability computation using entropy based on isotropic level set-like propagation. Therefore, inter-subject variability of sulcal curves and sulcal depth defined in the template space could be straightforwardly integrated into the entropy estimation.

### Conflict of interest statement

The authors declare that the research was conducted in the absence of any commercial or financial relationships that could be construed as a potential conflict of interest.
